# Supplementary material for: Patterns of Intron Gain and Loss in Fungi
Source: PLoS Biol. 2004 Nov 30;2(12):e422. doi: 10.1371/journal.pbio.0020422 (PMC532390; doi:10.1371/journal.pbio.0020422)
Supplement: Table S1 — Also available at http://genes.mit.edu/NielsenEtAl/. (4.3 MB ZIP). [file pbio.0020422.st001.zip › NielsenEtAl/html/90.html]

AN6126.1.NCU08535.1.MG07613.1.FG06580.1


```
 CLUSTAL W (1.82) Multiple Sequence Alignments - Introns Inserted


Sequence 1: NCU08535.1	2275 aa
Sequence 2: FG06580.1	2271 aa
Sequence 3: MG07613.1	2344 aa
Sequence 4: AN6126.1	2288 aa
Alignment Length: 2369 aa
Number Identitical Residues: 1387 aa
Alignment Score (without introns) 64525


MG07613.1 	MTETNGTAAAANSSRQRNGANGVTVPVANGKATYAQR~HKIADHFIGGNRLENAPPSKVK
NCU08535.1	MADTTA-SDAVVQAS------------------YAAK~HNLAPHFIGGNRLENAPPSKVK
FG06580.1 	MTEISAGAQEGANGR------------------SVPY0FNIADHFIGGNRLANAPSGKVK
AN6126.1  	MGVPDGTTNGHGGSR-------------------AAK~HNLPSHFIGGNHLDAAAPSSVK
          	*    . :     .                    .   .::. ******:*  *....**

MG07613.1 	EWVAAHDGHTVITN0VLIANNGIAAVKEIRSVRKWAYETFGDERAIQFTVMATPEDLQAN
NCU08535.1	DFVASHDGHTVITN0VLIANNGIAAVKEIRSVRKWAYETFGDERAIKFTVMATPEDLQAN
FG06580.1 	DFVAQNDGHTVITN0VLIANNGIAAVKEIRSVRKWAYETFRDERAIHFTVMATPEDLQAN
AN6126.1  	DFVANHEGHSVITS0VLIANNGIAAVKEIRSVRKWAYETFGNERAIQFTVMATPEDLAAN
          	::** ::**:***. ************************* :****:********** **

MG07613.1 	ADYIRMADHYVE~VPGGTNNNNYANVELIVDVAERMNVHAVWAGW~GHASENPKLPESLA
NCU08535.1	ADYIRMADHYVE~VPGGTNNHNYANVELIVDIAERMDVHAVWAGW2GHASENPKLPESLA
FG06580.1 	AEYIRMADHYVE~VPGGTNNHNYANVELIVDIAERMNVHAVWAGW~GHASENPKLPESLA
AN6126.1  	ADYIRMADQYVE0VPGGTNNNNYANVELIVDVAERMDVHAVWAGW~GHASENPRLPESLA
          	*:******:*** *******:**********:****:******** *******:******

MG07613.1 	ASPKKIIFIGPPGSAMRSLGDKISSTIVAQHAQVPCIPWSGTGVDAVQIDKKGIVTVDDD
NCU08535.1	ASPKKIVFIGPPGSAMRSLGDKISSTIVAQHADVPCIPWSGTGVSEVKVDDNGIVTVPDD
FG06580.1 	ASPNKIVFIGPPGSAMRSLGDKISSTIVAQHAAVPCIPWSGTGVDQVAVDDKGIVTVADD
AN6126.1  	ASPKKIIFIGPPASAMRSLGDKISSTIVAQHAQVPCIPWSGTGVDEVKVDENGIVTVEEE
          	***:**:*****.******************* ***********. * :*.:***** ::

MG07613.1 	TYAKGCVTSWQEGLEKARQIGFPVMIKASEGGGGKGIRKAVSEEGFEELYKAAASEIPGS
NCU08535.1	VYLKGCVSSWQEGLEKAREIGFPVMIKASEGGGGKGIRKVLNEDNFESLYNAAASEIPGS
FG06580.1 	IYAQGCVTSWEEGLEKAKEIGFPVMIKASEGGGGKGIRKATEEEGFEALYKAAASEIPGS
AN6126.1  	VYNKGCTFSPEEGLEKAKQIGFPVMIKASEGGGGKGIRKVEKEEDFINLYNAAANEIPGS
          	 * :**. * :******::********************. .*:.*  **:***.*****

MG07613.1 	PIFIMKLAGNARHLEVQLLADQYGNNISLFGRDCSVQRRHQKIIEEAPVTIAKPDTFKAM
NCU08535.1	PIFIMKLADSARHLEVQLLADQYGNNISLFGRDCSVQRRHQKIIEEAPVTIAKPMTFKAM
FG06580.1 	PIFIMKLAGNARHLEVQLLADQYGNNISLFGRDCSVQRRHQKIIEEAPVTIAKPDTFKAM
AN6126.1  	PIFIMKLAGNARHLEVQLLADQYGNNISLFGRDCSVQRRHQKIIEEAPVTIANPTTFQAM
          	********..******************************************:* **:**

MG07613.1 	EEAAVRLGRLVGYVSAGTVEYLYSHADDKFYFLELNPRLQVEHPTTEGVSGVNLPASQLQ
NCU08535.1	EEAAVRLGRLVGYVSAGTVEYLYSHADDKFYFLELNPRLQVEHPTTEMVSGVNLPAAQLQ
FG06580.1 	EDAAVRLGKLVGYVSAGTVEYLYSHSDDKFYFLELNPRLQVEHPTTEMVSGVNLPAAQLQ
AN6126.1  	ERAAVSLGKLVGYVSAGTVEYLYSHADDKFYFLELNPRLQVEHPTTEMVTGVNLPAAQLQ
          	* *** **:****************:********************* *:******:***

MG07613.1 	IAMGIPLHRISDIRLLYGVDPKLSTEIDFDFKNPDSEKTQRRPSPKGHLTACRITSEDPG
NCU08535.1	VAMGIPLHRIRDIRLLYGVDPRTASEIDFEFKNPESEKTQRRPTPKGHTTACRITSEDPG
FG06580.1 	IAMGIPLHRIRDIRLLYGVDPKTSSDIDFEFKSEDTSSSQRRPQPKGHTTACRITSEDPG
AN6126.1  	IAMGIPLHRIRDIRLLYGVDPNTSAEIDFDFSSEESFKTQRRPQPKGHTTACRITSEDPG
          	:********* **********. :::***:*.. :: .:**** **** ***********

MG07613.1 	EGFKPSNGVMHELNFRSSSNVWGYFSVGTQGGIHSFSDSQFGHIFAYGENRSASRKHMVI
NCU08535.1	EGFKPSNGVLHDLNFRSSSNVWGYFSVGSAGGIHSFSDSQFGHIFAYGENRAASRKHMVV
FG06580.1 	EGFKPSNGVMHELNFRSSSNVWGYFSVSSQGGIHSFSDSQFGHIFAYGENRSASRKHMVM
AN6126.1  	EGFKPSSGTMHELNFRSSSNVWGYFSVGTAGGIHSFSDSQFGHIFAYGENRSASRKHMVI
          	******.*.:*:***************.: *********************:*******:

MG07613.1 	ALKELSIRGDFRTTVEYLIKLLETEAFEENTITTGWLDELISKKLTAERPDKMLAVVSGA
NCU08535.1	ALKELSIRGDFRTTVEYLIKLLETEAFEENTITTGWLDELISKKLTAERPDPILAVVCGA
FG06580.1 	ALKELSIRGDFRTTIEYLIKLLETEAFEDNTISTGWLDELISKRLTAERPETMLAVTCGA
AN6126.1  	ALKELSIRGDFRTTIEYLIKLLETPAFEENKITTGWLDQLISNKLTAERPDTTIAVLCGA
          	**************:********* ***:*.*:*****:***::******:  :** .**

MG07613.1 	VTKAHLASEQCLNEYKAGLEKGQVPSKDILKTTFPIEFIYEGERYRFTVTRSSKDSYHLF
NCU08535.1	VTKAHIASEGCMTEYRAGLEKGQVPSKDILKTVFPVDFIYEGYRYKFTVTRSSADSYHLF
FG06580.1 	VTKAHIASEACMTEYRAGLEKGQVPSKDVLKTVFTIDFIYEGFRYKFTATRASVDSYHLF
AN6126.1  	VTKAHQASEARLEEYRNGIQKGQVPSKDVLKTVFPVDFIYEGKRYKFTATRAGLDSYHLF
          	***** ***  : **: *::********:***.*.::***** **:**.**:. ******

MG07613.1 	INGSNCSVGVRPLSDGGLLILLDGKSHNVYWKEEVGATRISIDNRTCLLEQENDPTQLRT
NCU08535.1	INGSKCTVGVRALSDGGLLILLNGRSHNVYWKEEVAATRMSVDSKTCLLEQENDPTQLRT
FG06580.1 	INGSKCHVGVRSLSDGGILVLLDGRSHSVYWKEEVGATRLSVDSKTCLLEQENDPTQLRS
AN6126.1  	INGSKCSIGVRALADGGLLVLLNGRSHNVYWKEEAAATRISVDGKTCLLEQENDPTQLRT
          	****:* :***.*:***:*:**:*:**.******..***:*:*.:**************:

MG07613.1 	PSPGKLVKYTVESGDHIKAGQTFAEVEVMKMYMPLVAQEDGTVQLIKQPGSTLEAGDILG
NCU08535.1	PSPGKLVKYTVENGEHVSAGQTFAEVEVMKMYMPLIAQEDGIVQLIKQPGATLEAGDILG
FG06580.1 	PSPGKLVKYSVENGAHVRAGQAFAEVEVMKMYMPLLAQEDGVVQLIKQPGATLEAGDILG
AN6126.1  	PSPGKLVKFTVENGEHVRAGQPFAEVEVMKMYMPLIAQEDGIVQLIKQPGATLEAGDILG
          	********::**.* *: ***.*************:***** ********:*********

MG07613.1 	ILSLDDPSRVKQAQPFLNKLPEYGNPVITGNKPAQRFTQLHNILDNIMLGYDNTTVMRDS
NCU08535.1	ILALDDPSRVKQAQPFLGQLPEFGAPVVVGSKPAQRFRLLYDTLQNILMGYDNQIIMQQT
FG06580.1 	ILALDDPSRVKQAQAFVDKLPEYGEPVVVGAKPAQRFSLLYNILHNILLGYDNSVIMDNT
AN6126.1  	ILALDDPSRVKHAQPFTEQLPPIGPPQVVGNKPAQRFFLLHSILENILKGFDNQVIMNST
          	**:********:**.*  :**  * * :.* ******  *:. *.**: *:**  :* .:

MG07613.1 	LLQLIEVMRDPDLPYSEWNSQFSALHTRMPQKLEAQLSHVIDRARARQSEFPAKALKKTF
NCU08535.1	LKDLIEVLRDPKLPYSEFTAQFSALHARMPQKLDAQLTQVLEKASSRSAEFPARNLGKVF
FG06580.1 	LKELIEVLRDTELPYSEWNAQFSALHSRMPQKLDAQFTQIIERSKSRNADFPAKALSKAL
AN6126.1  	LKELIEVLRDPELPYSEWNAQSSALHSRMPQKLDAQLQNIVDRARSRKAEFPARQLQKTM
          	* :****:**..*****:.:* ****:******:**: :::::: :*.::***: * *.:

MG07613.1 	HKFIDEHVARQ-DAGLLKTTLAPLVDVLDLYEEGQKAHELKTMSNLIAKFIETEKLFSGR
NCU08535.1	QKFLDENVASKVDAELLKTTLAPLTTVIDQYSEGQKVHELNVIRDLLTSYVEVERLFSGR
FG06580.1 	HKFIEDNVDAG-DAGILKTTLAPLTEIIDMYIEGQRSRELNVIKSILEQYYEVEALFMNQ
AN6126.1  	VRFIEENVNPA-DAEILKTTLLPLVQVINNYIEGLKAHEYKVFVGLLEQYYAVEKLFSGS
          	 :*::::*    ** :***** **. ::: * ** : :* :.: .:: .:  .* ** . 

MG07613.1 	RLQDDDVILKLRDENKDDIQKVVQTVLSHSRVPTKSLLVLAIIEEYRPNKPDVGNVTKYF
NCU08535.1	RLQDEEVILKLRDENKEDIKKVTQTVLSHSRVAAKNSLILAILDEYRPNKPNVGNVSKYL
FG06580.1 	-PQEDAVILQLRDQHKDDIMKVVHTVLSHSRVSSKSSLILAILEEYRPNKPKAGNVAKSL
AN6126.1  	KARYEDGILALREEHKDDVATIVQIALSHSRIGAKNDLILAILSIYRPNQPGMANVGQYF
          	  : :  ** **:::*:*: .:.: .*****: :*. *:***:. ****:*  .** : :

MG07613.1 	RDVLKKLTELESRLTAKVSIKAREVLIQCSLPSLDERKAQMEHILRSSVVESRYGEAAYD
NCU08535.1	RPVLRKLAELESRQTAKVSLKAREILIQCALPSLEERTAQMEHILRSSVVESRYGETGWD
FG06580.1 	RETLRLLTELQSRHTSKVSLKAREIMIQCALPSLEERTSQMEHILRSSVVESRYGETGWD
AN6126.1  	KSILKKLTEIESRAAAKVTLKAREVLIQCALPSLEERLSQMELILRSSVAESQYGETGWA
          	:  *: *:*::** ::**::****::***:****:** :*** ******.**:***:.: 

MG07613.1 	HRFPNMDVIKEVVDSKYTVFDVLSLFWAHSDKWVSLAALEVYIRRAYRAYDLGKIDYQYD
NCU08535.1	HREPSLDIIKEVVDSKYTVFDVLTLFFAHEDPWVSLAALEVYVRRAYRAYVLKKIEYHTD
FG06580.1 	HREPSLDIIKEVVDSKYTVFDVLTMFFAHDDPWVSLASLEVYVRRAYRAYLLKQIEYHQD
AN6126.1  	HREPDLGALKEVVDSKYTVFDVLPRFFVHKDAWVTLAALEVYVRRAYRAYSIQGIQYHHE
          	** *.:. :**************. *:.*.* **:**:****:******* :  *:*: :

MG07613.1 	EEDDPVFVSWDFVMKLSGDVEFGIGGPLQSAAPSSPATPNEF--TFNRVTSLSDMSYLAK
NCU08535.1	ETETPSFLSWDFSLRKLGHSEFGL--PIQSAAPSTPGTPVDS--TFKRISSISDMSYLSH
FG06580.1 	ENDNPQFVSWDFQLRKLGQSEFGL--PLQSAAPSTPATPSGGEFNFKRIHSISDMSYLNS
AN6126.1  	GE--PAFLSWDFTMGKLGQPEFGS--MTAVTHPSTPSTPTTESNPFKRVSSISDMSNLLN
          	    * *:**** :   *. ***       : **:*.**   .  *:*: *:**** *  

MG07613.1 	KTAEQPIRKGVMVACKFFDDVDDDLPKALESLPMKGKLKRLGSSGPAAAIASQLDNKRRP
NCU08535.1	KTQDEPTRKGVIIPCKFLDDADELLSRALEKLPVLGARKRN-------SVIPDLNEKRRP
FG06580.1 	KWEDEPTRKGVIVPCKYIDEAEDLIGKALEALAHEQKQKKKNTP----GLIPDLSGKRKP
AN6126.1  	DSPNGTPRKGVILPVQYLEDAEEYLTKALEVFPRAGTRKPSDHG-----LIASLEGKRRP
          	.  : . ****::. :::::.:: : :*** :.     *  .       : ..*. **:*

MG07613.1 	AAASRDD--DELTAVVNVAIRDAESRDDDETLSRIKPLLASFKEELCARRVRRITFICGK
NCU08535.1	-PLQRLETFDELSAVVNVAVRDAEGRSDEEILKEILPLVHQHREDLFARRVRRITFVCGR
FG06580.1 	APLKQQE--EELSAVINVAIRDVESRDDREALEDILPIVEQYKDELLARGVRRLTFICGH
AN6126.1  	APRADSES-TELTGVLNIAIRDIEELDDAQIVAQISKLVSSFKDEFLARRIRRVTFICGK
          	:.    ::  **:.*:*:*:** *  .* : :  *  :: ..:::: ** :**:**:**:

MG07613.1 	NDASYPAYYTFRGPNYDEDESIRHSEPALAFQLELDRLSKFKYKSVFTENKNIHVYEAIG
NCU08535.1	NDGSYPGYFTFRGPEYVEDDSIRHSEPALAFQLELGRLSKFKIKPVFTENKNIHVYEAIG
FG06580.1 	SDGSYPGYYTFRGPEYKEDDSIRHSEPALAFQLELARLSKFNIKPVFTENKSIHVYEGIG
AN6126.1  	-DGVYPSYYTFRGPNYEEDESIRHSEPALAFQLELNRLSKFKIKPVFTENRNIHVYEAIG
          	 *. **.*:*****:* **:*************** *****: *.*****:.*****.**

MG07613.1 	RGVDT----DKRYFTRAVIRPGRLRDEIPTAEYLISEADRVVNDIFDALEIIGNNNSDLN
NCU08535.1	KGVET----DKRYFTRAVIRPGRLRDEIPTAEYLISEADRVINDIFDALEIIGNNNSDLN
FG06580.1 	KSVDT----DKRYFTRAVIRPGRLRDEIPTAEYLISEADRVVNDIFDALEIIGNNNSDLN
AN6126.1  	KGPENDKALDKRYFVRAVVRPGRLRDDIPTAEYLTSEADRLMNDILDALEVIGNNNSDLN
          	:. :...: *****.***:*******:******* *****::***:****:*********

MG07613.1 	HLFLNFIPVFNIHPGEVEHALAGFLERFGSRGWRLRVSQVEIRIICTDQNSGAKLPIRCY
NCU08535.1	HMFLNFTPVFQLQPEEVEHSLQGFLDRFGPRGWRLRVAQVEIRIICTDPATGMPYPLRVI
FG06580.1 	HVFINFSPVFQLQPKEVEESLQGFLDRFGIRAWRLRIAQVEIRIICTDPQTGEPYPLRVV
AN6126.1  	HIFINFSPVFNLQPKDVEEALAGFLDRFGLRLWRLRVTGAEIRILCTDPATGMPYPLRVI
          	*:*:** ***:::* :**.:* ***:*** * ****:: .****:***  :*   *:*  

MG07613.1 	INNTSGFVVKVELYHERRNDKGDLILHYISGTSKTGALHLLPANTPYPTKNWLQPKRYKA
NCU08535.1	ITNTSGYVIQVELYAERKSEKGEWVFHSIGGTTKIGSMHLLPVNTPYPTKNWLQPKRYKA
FG06580.1 	ITNTSGYVVDVDMYAERKSEKGQWVFHSIGGTHEKGPMHLMPVSTPYAIKNWLQPKRHDA
AN6126.1  	ISNTVGYIIQVELYIEKKSEKGEWLLHSIGGTNKLGSNHLRPVSTPYPTKEWLQPKRYKA
          	*.** *:::.*::* *::.:**: ::* *.** : *. ** *..***. *:******:.*

MG07613.1 	HLIGTQYVYDFPELFRQAIQLSWKKLEAQHSPIASKKPEEGECTHTLELVLDDQDNLQAI
NCU08535.1	HLMGTQYVYDFPELFRQAIQNSWVKAVRMDSSLADKQPPVGECIEFSELVLDDHDNLIEV
FG06580.1 	HGMGTQYVYDFPELFRQAIQNTWNKAVKAQPSLASQQPKTGDCISFTELVLDDKDNLDEV
AN6126.1  	HVMGTQYVYDFPELFREAFQNSWTKAIEKSPSLIERRPPLGECMEYSELVLDDTDNLVEI
          	* :*************:*:* :* *     ..: .::*  *:*    ****** ***  :

MG07613.1 	EREPGTNTCGMVGWIITAFTPEYPSGRRFVVVANDITYSIGSFGPKEDHFFNKCTEYAQK
NCU08535.1	SREPGTNTCGMVGWLIRARTPEYPNGRKFVVVANDITFNIGSFGPKEDNFFFKCTELARK
FG06580.1 	NREPGTNTCGMVGWIFRARTPEYPNGRRFIVIANDITYKIGSFGPKEDDFFYKCTELARK
AN6126.1  	SRGPGTNTHGMVGWIVTARTPEYPEGRRFIIVANDITFQIGSFGPLEDKFFHKCTELARK
          	.* ***** *****:. * *****.**:*:::*****:.****** **.** **** *:*

MG07613.1 	LGIPRIYLSANSGARLGVANELIPHFKVAWTDPSNPNKGFDYLYLLEEAKKKFEET----
NCU08535.1	LGIPRIYLSANSGARLGLATELMPHFSVAWNDPSKPEAGFKYLYLDDAGKKRFENT----
FG06580.1 	LGIPRIYLSANSGARLGLADELMGHFKVAWNNPEKQDAGFKYLYLDDETKTRFEKD----
AN6126.1  	LGIPRVYLSANSGARIGMADELIPYFSVAWNDPAKPEAGFKYLYLTPEVKKKFDASKQKE
          	*****:*********:*:* **: :*.***.:* : : **.****    *.:*:  ....

MG07613.1 	VITEEITVDGETRHKIVTIVGAEDGLGVECLRGSGLIAGATSRAYNDIFTCTLVTCRSVG
NCU08535.1	VITEEITEGDEKRHKIVTIVGAEDGLGVECLRGSGLIAGATSRAYNDIFTCTLVTCRSVG
FG06580.1 	VITEEVTEDGEKRHKIVTVVGKEEGLGVECLRGSGLIAGATSRAYNDIFTVTLVTCRSVG
AN6126.1  	VITELIHDEGEERHKITTIIGAKDGLGVECLKGSGLIAGATSRAYEDIFTITLVTCRSVG
          	**** :   .* ****.*::* ::*******:*************:**** *********

MG07613.1 	IGAYLVRLGQRAVQIEGQPIILTGAPALNNLLGREVYTSNLQLGGTQIMYRNGVSHMTAN
NCU08535.1	IGAYLVRLGQRAVQIEGQPIILTGAPALNNVLGRQVYTSNLQLGGTQIMYRNGVSHLTAN
FG06580.1 	IGAYLVRLGQRAVQIEGQPIILTGAAALNNLLGREVYTSNLQLGGTQIMHRNGVSHMTAN
AN6126.1  	IGAYLVRLGQRAIQVEGQPIILTGAPAINKLLGREVYTSNLQLGGTQIMYRNGVSHMTAA
          	************:*:**********.*:*::***:**************:******:** 

MG07613.1 	DDMEGIAKIMEWMSFVPDQRNRPVPIMAPLDKWDREVDFTPPQRQPYDPRWLIAGKEDAD
NCU08535.1	DDFAGVSKIVEWMSFVPDKRNNPVPISVSVDTWDRDVVYTPPQKQPYDVRWMIGGKEDEN
FG06580.1 	DDFAGVSKIVEWMSFVPEKRNSPVPVSPSVDSWDRDVTYYPPQKQPYDVRWLIGGREGEN
AN6126.1  	NDFDGVEKIVDWLAFVPEKKGSLPPIRPLADPWDRDVSYHPPAKQAYDVRWLINGKEDEE
          	:*: *: **::*::***:::.   *:    * ***:* : ** :*.** **:* *:*. :

MG07613.1 	GFQSGLFDRGSFVETLGGWARTVVVGRARLGGIPMGVIAVETRAVENITPADPANADSME
NCU08535.1	GYQPGLFDKDSFVETLGGWARTVVVGRARLGGIPMGVIAVETRSVENITPADPANPDSIE
FG06580.1 	GFESGLFDKDSFVEALGGWAKTVVVGRARLGGIPMGVIGVEVRSVENITPADPANPDSIE
AN6126.1  	GFLPGLFDAGSFEEALGGWARTVVVGRARLGGIPMGVIAVETRSVENVTPADPANPDSME
          	*: .**** .** *:*****:*****************.**.*:***:*******.**:*

MG07613.1 	QVTNEAGGVWYPNSAFKTAQAINDFNNGEQLPLMILANWRGFSGGQRDMYNEVLKYGSFI
NCU08535.1	QVANEAGGVWYPNSAFKTAQAINDFNNGEQLPLMILANWRGFSGGQRDMYNEVLKYGSFI
FG06580.1 	QVSNEAGGVWYPNSAFKTAQAINDFNNGEQLPLMILANWRGFSGGQRDMYNEVLKYGSFI
AN6126.1  	MITQEAGGVWYPNSSFKTAQALRDFNNGEQLPVMILANWRGFSGGQRDMYNEVLKYGSYI
          	 :::**********:******:.*********:*************************:*

MG07613.1 	VDALVKYEQPIFVYIPPHGELRGGSWVVVDPTINSTVMEMYADKEARGGVLEPEGIIGIK
NCU08535.1	VDALVKFEQPVFIYIPPFGELRGGSWVVVDPTINPVAMEMYADVDARGGVLEPEGIIGIK
FG06580.1 	VDALVKYEQPIFIYIPPFGELRGGSWVVVDPTINPTAMEMYADTEARGGVLEPEGMIGIK
AN6126.1  	VDALVKYEQPIFVYIPPFGELRGGSWVVVDPTINPDQMEMYADEEARGGVLEPEGIVNIK
          	******:***:*:****.****************.  ****** :**********::.**

MG07613.1 	YKKEKQLETMARLDPVYGELKKRMLAVQAAAKDKTGAADKEELEQIKKQMTQREEQLGPI
NCU08535.1	YRKDKQLETMARLDPVYADLKR---------QSADASLPKEESDAIKQKMTEREQQLLPV
FG06580.1 	YRKQKQIQTIIRMDPTYAGLKK---------QLEDSSLSSEQTDEIKKKMAAREKELLPV
AN6126.1  	FRRDKQLETMARLDPTYGELRR---------ALQDKNLSKEKLSDIKDKMAAREEQLLPV
          	::::**::*: *:**.*. *::                 .*: . **.:*: **::* *:

MG07613.1 	YQQIAIQFADLHDRAGRMKAKGTIREVIEWKEARRFFYWRVRRRINEEYLIRKMARATAG
NCU08535.1	YAQISVQFADLHDRAGRMKAKGVIREVLEWQNARRFFYWRVRRRLNEEYILRRIISATTP
FG06580.1 	YSQIALQFADLHDRAGRMKAKGAIRDVLEWSSSRRFFYWRLRRRLNEEYILRRMTSTIIS
AN6126.1  	YMQIALQFADLHDRAGRMQAKNTIRQALSWKNARRFFYWRVRRRISEEYIIKRMLTACPA
          	* **::************:**..**:.:.*..:*******:***:.***:::::  :   

MG07613.1 	---------LSKTTSAKISESARKGYLRSLAAWTAVPD--FDHEDRKVATWYEENTKIVE
NCU08535.1	GG------APSKALTASSTKER-ARHLQLLQAWSGIEK--FDTADREVAVWYEENRHLVQ
FG06580.1 	---------TSHNQTATKDKETRDKYLHLLRSWSAIVD--WEKDDQAVTEWYETERKAIG
AN6126.1  	PVQGEGSGAVAQGVSPAPSDSPRTTHLRTLHSWTPFLENEVENDDRRVAVWYEENKELIQ
          	  ....:.: ::  :.   ..    :*: * :*: . ... :  *: *: *** : . : 

MG07613.1 	EKLKALKTDAVASEISALIRGGTWTHEEVETPSTPSIEKSKAPLEESTEPRSPTGPVPTT
NCU08535.1	TKIDALKAEAITTEMRDLIRA-------------------------------------AS
FG06580.1 	EKVDALKSEVLAAEVASVVRG-------------------------------------HA
AN6126.1  	EKIEALKSQAIASQISDVLFS---------------------------------------
          	 *:.***::.:::::  :: .                                       

MG07613.1 	ENGLSSSWKGVRDVLRTMPVEDRDKILQFLKE-
NCU08535.1	KGSDDAAWKGVRDILSVMPVEEREKVVKYLTTL
FG06580.1 	K----AGWTGVREVMRVMPVEEREQILKYLQQ-
AN6126.1  	--NRESGLKGIQQALSFLPVEEKESILKYLGSN
          	  . .:. .*::: :  :***:::.::::*
```
